# A cross-sectional survey of the readiness of consumers to adopt an environmentally sustainable diet

**DOI:** 10.1186/s12937-020-00644-7

**Published:** 2020-12-09

**Authors:** Amy Culliford, Jane Bradbury

**Affiliations:** Faculty of Health and Social Care, Edge Hill University, Ormskirk, UK

**Keywords:** Public health, Nutrition, Sustainable diets, Dietary guidelines, Environment, Behaviour change, Stage of change, Transtheoretical model, Plant-based diets

## Abstract

**Background:**

The current food system is responsible for significant environmental damage therefore, encouraging consumers to adopt an environmentally sustainable diet is a key public health challenge. Dietary guidelines have been developed that outline recommendations for purchasing and consuming food in an environmentally sustainable manner, but they have not yet been incorporated in UK national dietary guidelines.

**Methods:**

Via an online survey of UK adults, we evaluated consumers’ perceptions of the environmental benefit of various sustainable diet recommendations, their readiness to adopt these behaviours using the stage of change construct of the Transtheoretical Model, the factors that influenced their food choices, and their current consumption of plant- and animal-based sources of protein. Additionally, we investigated how demographic characteristics and food choice motives were associated with perceived environmental benefit of and readiness to adopt these sustainable diet recommendations.

**Results:**

The survey was completed by 442 participants (66% female, 80% aged 25–54 years, 85% with higher education). The majority of participants considered the recommendations to ‘reduce consumption of air-freighted foods’ (79%), ‘reduce food waste’ (75%), and ‘buy locally grown produce’ (78%) to have a high environmental benefit, whereas a smaller proportion of participants perceived ‘prioritise plant-based proteins’ (42%) and ‘choose organic produce’ (27%) to have a high environmental benefit. Differences in perceptions and readiness to adopt sustainable dietary behaviours were observed between demographic groups, with women significantly more likely than men to be in action/maintenance (A/M) stages of change for prioritising plant proteins (OR 0.54), and younger participants more likely to be in pre-contemplation/contemplation (PC/C) stages of change for ‘choose organic produce’ (OR 2.03) and ‘choose sustainable fish’ (OR 2.45). Health, cost, environmental sustainability and taste were the most commonly reported food choice motives. Reporting environmental sustainability as a food choice motive was associated with readiness to adopt sustainable diet recommendations.

**Conclusions:**

We found that consumers in the UK are engaged with some aspects of sustainable diets but remain resistant to others. The results of this study indicate that acceptable dietary guidelines could be developed to address environmental sustainability. Several behaviours were identified that consumers were willing to adopt, but there were barriers preventing them, highlighting that policy action is required to enable behaviour change to occur. Differences between demographic groups highlight potential targets for future campaigns promoting sustainable diets.

**Supplementary Information:**

The online version contains supplementary material available at 10.1186/s12937-020-00644-7.

## Background

Developing a sustainable food system to feed the growing global population is one of the major challenges of the 21st century [1]. Agricultural food production is responsible for 70% of water use and 30–35% of greenhouse gas (GHG) emissions, which contribute to global warming [2]. The current food system is a key driver of environmental degradation through loss of biodiversity, deforestation and pollution [3] and the effects of climate change and environmental damage are also likely to increasingly challenge food security over the next century [4]. Shifting towards a more sustainable food system is therefore paramount to achieving several of the United Nations Sustainable Development Goals, particularly goal two “End hunger, achieve food security and improved nutrition and promote sustainable agriculture” and goal 13 “Take urgent action to combat climate change and its impacts” [5].

The UK government has pledged to reduce total GHG emissions to 20% of 1990 levels over the next 30 years [6, 7]. It is estimated that food consumption is responsible for 19–27% of UK GHG emissions, with more than half these arising from agricultural production, and the remainder from other aspects of the life cycle including transport, retail and marketing [8]. It is estimated that through realistic changes, UK consumers could reduce their dietary GHG emissions by 25% [9], which is equivalent to 5–7% of total UK GHG emissions. Combining individual dietary changes with industrial improvements, such as use of renewable energy and advances in agricultural technology, could result up to 70% reduction in food-related GHG emissions [2]. Adopting a sustainable diet also contributes to improvements in other environmental indicators such as biodiversity, land and water use, however further research is needed to enable these factors to be measured and incorporated appropriately [10].

The Food and Agriculture Organisation (FAO) define sustainable diets as “those diets with low environmental impacts which contribute to food and nutrition security and to healthy life for present and future generations” [11], therefore promoting sustainable diets is also an opportunity to improve population health outcomes. A significant body of research exists outlining the concept of a sustainable diet [3, 9, 12–14]. In these studies, dietary environmental impact is measured in terms of GHG emissions [10], and healthy diets are defined as those which meet existing nutrient intake guidelines and include food groups such as fruits, vegetables and whole grains which are associated with prevention of non-communicable diseases [12]. It is important to note that although there is significant overlap between health promoting and environmentally sustainable diets, they are not mutually inclusive as a nutritionally adequate diet can have a high environmental impact and a diet with low GHG emissions may be nutritionally deficient [15].

The overall environmental impact of an individual’s diet depends on what and how much they eat as well as where their food was grown and how it was processed [16]. A number of studies have concluded that animal products are the highest contributors to the dietary environmental footprint with the consensus that a shift towards plant-based diets is key to reducing GHG emissions and improving population health [8, 14, 17–21]. In particular, diets which eliminate red meat have a lower global warming potential [21] and reduced risk for non-communicable diseases such as heart disease, stroke and several types of cancer [22]. Scarborough et al. modelled several dietary patterns with the potential to reduce both GHG emissions and mortality rates in a UK population [23]. For example, replacing 50% of the meat and dairy in the current typical UK diet with fruit, vegetables and cereals resulted in a 19% decrease in GHG emissions and 36,910 fewer deaths from coronary heart disease, stroke or diet-related cancers [23].

Life cycle analysis (LCA) of various foods based on resource use, GHG emissions, and impact on soils have concluded that avoiding air-freighted foods, choosing organic over conventional produce, and reducing meat consumption are the diet-related behaviours which have the largest overall environmental benefit [24]. Furthermore, other dietary behaviours such as consuming local and seasonal produce, decreasing food and packaging waste, and consuming fish from sustainable sources can also help to reduce the impact of the food system on the environment [25].

These recommendations have been outlined in sustainable dietary guidelines developed by various international non-government organisations, including the European Public Health Association (EUPHA) [26] and the European Food Information Council (EUFIC) [27]. Incorporating sustainability aspects into national dietary guidelines is an important foundation for transforming the food system as these can shape food policies, marketing and labelling legislation, and encourage populations to adopt a more sustainable diet [28]. At present, UK national dietary guidelines (EatWell Guide) [29] do not explicitly include sustainability, although Steenson and Buttriss argue that eating a diet consistent with the EatWell Guide is likely to result in environmental as well as health benefits [30]. The Canadian government have recently published their Food Guide [31], which uses a similar plate model to the UK EatWell Guide, and includes guidelines which overlap significantly with sustainable diet recommendations developed by the British Dietetic Association (BDA) [25].

Fisher and Garnett reviewed existing dietary guidelines from several countries and concluded that sustainable diet guidelines should be accessible but ambitious [32]. In order to develop acceptable dietary guidelines that also address environmental sustainability, governments should take into account current consumption patterns, social norms and other cultural factors [33, 34]. It is therefore important to understand the current perception and level of engagement with sustainable diet recommendations in the UK before incorporating sustainability into national dietary guidelines. Despite extensive research into the environmental benefit of adopting sustainable diets, there is limited research on how acceptable these behaviour changes are to consumers.

We identified several studies that examined attitudes and intentions towards adopting a plant-based diet [35–37] or sustainable dietary habits [38–41], which concluded that plant-based diets are generally perceived by consumers to be beneficial for health and the environment [35, 37, 40]. However, reducing meat consumption is perceived to have a small environmental benefit compared with other behaviours such as reducing food and packaging waste [38, 39, 41, 42]. A small-scale study of UK consumers highlighted a willingness to reduce meat consumption by up to 20% but a reluctance to eliminate meat from the diet or to limit food choices to only those which are in season [43]. To our knowledge there has been no comprehensive study of engagement with sustainable diet recommendations amongst a UK population. Therefore, this study aimed to evaluate the perceived environmental benefit of a range of sustainable dietary recommendations and readiness to adopt these behaviours.

Persuading consumers to adopt sustainable diet recommendations is likely to be a challenge as adherence to health-related dietary guidelines is relatively low [44, 45]. Furthermore, the way we eat is influenced by many political, social and economic factors including price, availability and cultural traditions, as well as personal values such as taste and health [46, 47]. Allès, Péneau et al. [48] observed that health and taste are the factors which most influence individuals’ food choices and that environmental considerations were less important. An objective of the current study was to identify the primary food choice motives of consumers and to evaluate whether these factors influenced readiness to adopt sustainable diet recommendations.

Finally, perceptions and behaviours often cluster in sub-groups of populations and examining differences between demographic groups can identify potential targets for behaviour change interventions [49, 50]. Previous studies have reported conflicting findings in this area. For example, Tobler et al. concluded that females are more willing to adopt a plant-based diet and consume seasonal produce but found no significant differences between age groups or education level in a sample of Australian adults [39]. Conversely, another Australian study did not observe differences in willingness to adopt a plant-based diet between any demographic groups [35]. Therefore, this study aimed to analyse differences between demographic groups in perceived importance and readiness to adopt a plant-based diet as well as other sustainable diet recommendations amongst a UK population.

## Methods

### Study design

This research study employed a quantitative approach in the form of a cross-sectional, online survey of consumers’ perceived environmental benefit and readiness to adopt sustainable dietary recommendations. We aimed to expand on international research in this field in light of sustainable dietary guidelines recently published in the UK [25].

### Participants

Inclusion criteria for the study were that participants must be adults currently living in the UK. Children ≥18 years old were not eligible to take part. The study was promoted via the Environment Agency, a non-departmental public body with responsibilities relating to the protection and enhancement of the environment in England. However, participants were not required to be employees of the Environment Agency to take part in the study.

### Measures

A questionnaire was developed for the purposes of this study, based on a review of existing measures that have been used to assess public perceptions of sustainable dietary behaviours [35, 37, 39, 41]. Participants’ perceived environmental benefit of nine sustainable diet behaviours (avoid excess packaging; buy locally grown produce; consume seasonal fruits and vegetables; limit red and processed meat; prioritise plant proteins e.g. Quorn, beans, nuts, tofu; reduce consumption of air freighted foods; choose sustainable fish; reduce food waste; choose organic produce) was measured using a 5-point Likert scale from ‘very small benefit’ to ‘very large benefit’. The behaviours chosen were based on environmentally sustainable dietary guidelines published by the BDA [25].

Readiness to adopt these behaviours was measured using one question with six response options that corresponded with the stage of change construct of the Transtheoretical Model of behaviour change [51]. The stage of change construct provides a useful cross-sectional measure of an individual’s readiness to adopt a particular behaviour and has previously been used to measure readiness to adopt environment and health-related behaviours [38, 39] However, it is limited in that it does not measure actual behaviour and also does not provide information as to how or why an individual transitions between stages. The response options provided in the survey were: ‘I am not interested in doing this at the moment’ (pre-contemplation), ‘I am thinking about this but I need more information’ (contemplation), ‘I would like to do this but there are things stopping me’ (planning), ‘I have started to do this some of the time’ (action), ‘I am doing this confidently most of the time’ (maintenance), and ‘I am not currently doing this but have done in the past’ (relapse).

To understand the factors that influence participants’ dietary choices, they were asked to select the three most important food-choice motives from a provided list (health, cost, religion, taste, environmental sustainability, availability, animal welfare, and weight loss). Participants were also asked to report their typical weekly frequency of consumption of animal-based (white meat, red meat, processed meat, dairy products, eggs, and fish) and plant-based (beans/lentils, processed meat alternatives, non-dairy milks, and nuts/seeds) sources of protein. The socio-economic information collected was age group, gender, education level, geographic location, and whether participants had children living at home.

The data collection tool was pre-tested via cognitive interviews with five individuals from different demographic groups within the recruitment pool. Cognitive interviewing is a useful method to ensure that participants understand and interpret the questions as intended and the response options are appropriate [52]. This was particularly important as previous research suggests that many people are not familiar with the topic of sustainable diets [40, 53]. Modifications were made to the questionnaire as a result, including addition of the ‘relapse’ stage of change response option and listing specific examples of plant-based proteins in the food frequency questionnaire.

### Procedure

Written permission to recruit employees from the Environment Agency was obtained prior to data collection. An online survey tool, Smart Survey, was used to deliver the questionnaire. A self-recruit sampling method was utilised whereby a link to the online survey was shared via company e-newsletters and employees were invited to participate and to share the link with friends and family. The data collection period ran for a total of 6 weeks from July to August 2019. Ethical approval to conduct the study was granted by the appropriate Ethics Review Committee at Edge Hill University.

#### Statistical analysis

This study aimed to evaluate the perceived environmental benefit of sustainable dietary recommendations, readiness to adopt these behaviours, and differences in perceived importance and reported behaviours between demographic groups in a UK sample.

The data were exported from Excel to SPSS (IBM SPSS Statistics for Windows, Version 25.0. Armonk, NY: IBM Corp.) for statistical analysis. Due to small numbers of responses in several of the response categories, some variables were recoded into fewer response categories for the purposes of statistical analysis. Perceived environmental benefit was recoded into three categories by combining ‘very small’ and ‘small’ into ‘low perceived benefit’, ‘moderate’ as ‘medium perceived benefit’, and ‘large’ and ‘very large’ into ‘high perceived benefit’. The six stages of change were combined into three categories: pre-contemplation and contemplation (PC/C); preparation and relapse (P/R); action and maintenance (A/M). The stages were combined in this way to reflect groups of individuals who are not interested or may need further information (PC/C), those that experience other barriers (P/R) and those who are already taking action (A/M).

The gender variable was filtered to include only ‘female’ and ‘male’ responses; the age variable was recoded into two categories of ‘below 35 years’ and ‘35 years and over’; education level was recoded into ‘higher education’ and ‘secondary education and below’. Previous research has highlighted that generations born after 1982, particularly those with a higher education level, may be more aware of environmental sustainability than previous generations [54], therefore the variables were re-coded in this way to determine whether this applies to food-related issues.

The association between the perceived importance of environmentally sustainable behaviours and sociodemographic factors was assessed using Chi-square test for independence. Multinomial regression analysis was used to predict participants’ stage of change for ‘prioritise plant-based proteins’, ‘choose organic produce’, ‘choose sustainable fish’ and ‘limit red and processed meat consumption’ based on demographic variables and reported food-choice motives. Statistical significance was defined for all tests as *p* < 0.05.

## Results

A total of 442 participants completed the survey with all responses complete. Two thirds of the sample were women, most were aged between 25 years and 54 years old and educated to at least undergraduate degree level (Table 1).

**Table 1:** Demographics of sample in terms of number of participants (n) and percentage (%) of respondents

	n	%
**Gender**		
Female	292	66.1
Male	143	32.4
Other	3	0.7
**Age**		
18–24	24	5.4
25–34	129	29.2
35–44	105	23.8
45–54	117	26.5
55–64	65	14.7
65+	2	0.5
**Education**		
Post-graduate	204	46.2
Under-graduate	172	38.9
A-levels	46	10.4
GCSE	16	3.6
None	4	0.9
**Children**		
Yes	119	26.9
No	323	73.1

**Fig. 1:**
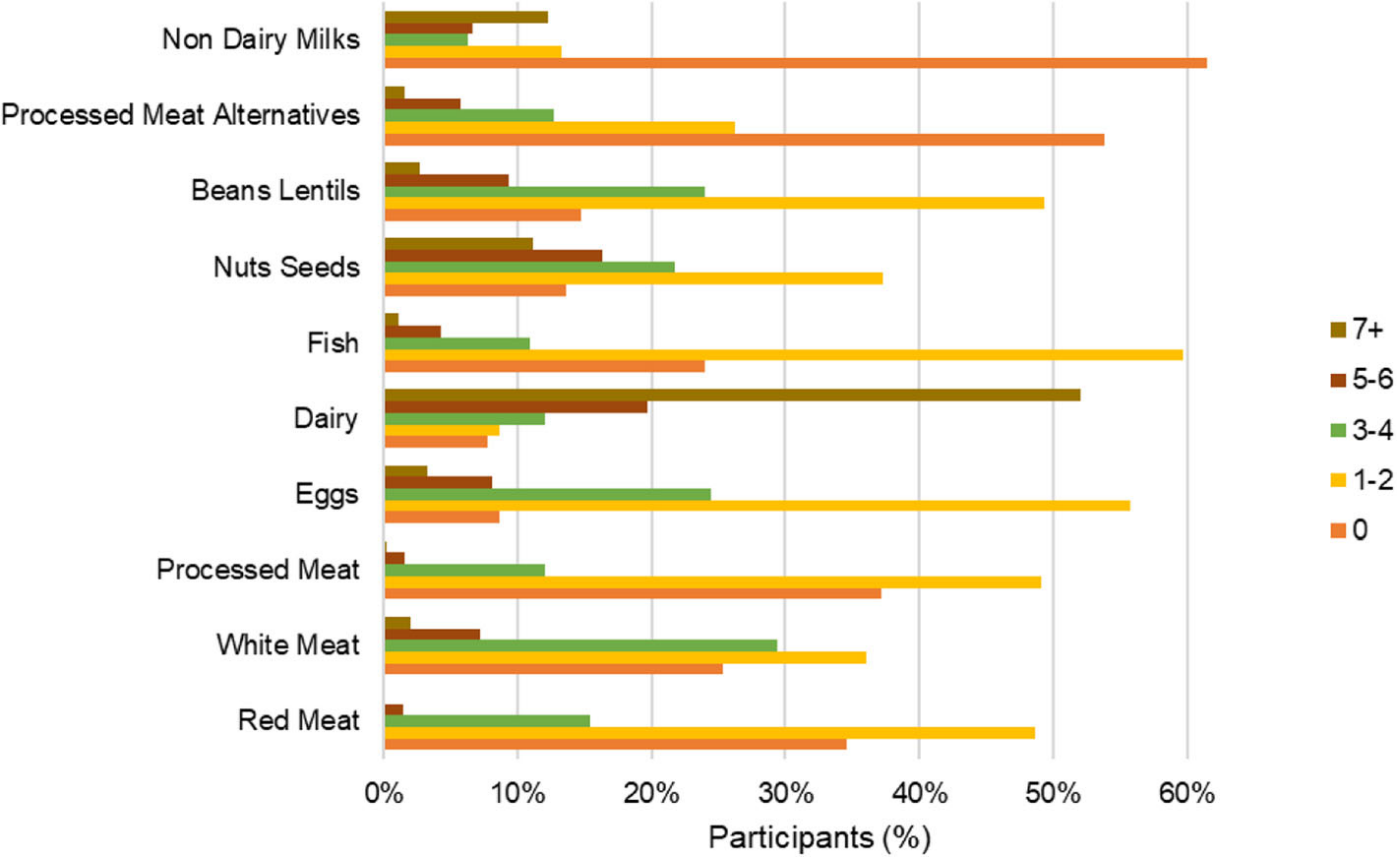
Weekly frequency of consumption of animal and plant-based proteins

The typical weekly frequency of consumption of animal and plant-based protein sources, ranging from never to at least once per day, is presented in Fig. 1. Remarkably, a third of participants reported that they never consume red or processed meat and a quarter do not consume white meat in a typical week. More than 50% reported consuming dairy products daily and eggs at least three times per week. Another interesting result is that the majority of participants reported consuming plant-based proteins sources such as nuts, seeds, beans and lentils at least once a week and more than 50% reported consuming processed plant-based meat and dairy alternatives at least once a week.

When participants were asked about their main food-choice motives, the top response was health (76%), followed by cost (55%), environment (53%) and taste (48%). Interestingly, animal welfare (31%) availability (20%), weight loss (12%) and religion (1%) were reported as the least influencing factors on participants’ food choices.

For seven of the nine sustainable diet recommendations studied, at least half of participants perceived a high environmental benefit. The only exceptions were ‘choose organic produce’ and ‘prioritise plant-based proteins’ which were perceived to have the lowest environmental benefit. ‘Buy locally grown produce’, ‘reduce consumption of air-freighted foods’ and ‘reduce food waste’ were perceived to have the highest environmental benefit of the recommendations studied (Table 2).

**Table 2:** Number (n) and percentage (%) of respondents rating low, medium or high environmental benefit

	Perceived Benefits					
	Low		Medium		High	
	n	%	n	%	n	%
Avoid excess packaging	38	9	113	26	291	66
Buy locally grown produce	20	5	78	18	344	78
Consume seasonal fruits and vegetables	19	4	107	24	316	72
Limit red and processed meat	47	11	102	23	293	66
Prioritise plant proteins	100	23	155	35	187	42
Reduce consumption of air freighted foods	15	3	77	17	350	79
Choose sustainable fish	46	10	113	26	283	64
Reduce food waste	24	6	87	20	331	75
Choose organic produce	160	36	163	37	119	27

The difference in perceived environmental benefit of sustainable dietary recommendations were explored by socio-demographic group age, children living at home, education level, and gender (Table 3). A larger proportion of participants with a higher education level associated a high environmental benefit with ‘limit red and processed meat’ (*p* <0.001), ‘prioritise plant-based proteins’ (*p* = 0.01) and ‘consume seasonal fruits and vegetables’ (*p* <0.001) compared to less educated participants. Having children living at home did not significantly affect perceived environmental benefit of sustainable diet recommendations.

**Table 3:** Chi-Square (X^2^) values for comparison of perceived environmental benefit between demographic groups

Perceived importance		Education				Children at home				Age				Gender			
		High (%)	Low (%)	X^2(1)^	P^(2)^	No (%)	Yes (%)	X^2^	p	U35 (%)	35 + (%)	X^2^	p	F (%)	M (%)	X^2^	p
Packaging	Low (%)	9	6	4.5	0.10	9	9	1.3	0.53	9	8	5.1	0.36	8	10	3.7	0.45
	Med (%)	27	17			27	23			28	22			23	30		
	High (%)	64	77			65	69			63	70			68	60		
Local	Low (%)	4	8	5.8	0.06	5	3	0.9	0.63	5	4	12.6	0.40	4	4	8.3	0.08
	Med (%)	19	11			18	16			20	15			14	24		
	High (%)	77	82			77	82			75	81			81	71		
Seasonal	Low (%)	3	11	12.1	< 0.001	4	4	1.0	0.60	4	4	10.1	0.88	3	6	9.7	0.05
	Med (%)	24	26			26	22			24	25			22	31		
	High (%)	73	63			70	74			72	71			75	64		
Red Meat	Low (%)	9	20	10.9	< 0.001	11	10	1.0	0.61	8	14	15.4	0.01	10	13	2.1	0.72
	Med (%)	22	29			22	25			20	27			22	25		
	High (%)	69	51			67	65			72	58			68	62		
Plant Protein	Low (%)	20	35	8.5	0.01	22	23	3.2	0.20	23	23	11.5	0.04	19	29	7.3	0.12
	Med (%)	35	35			33	42			30	42			35	36		
	High (%)	45	29			45	35			47	36			46	35		
Air Freighted	Low (%)	3	5	3.0	0.22	3	3	0.0	0.98	4	3	8.0	0.30	3	3	1.0	0.91
	Med (%)	16	23			18	17			20	14			16	20		
	High (%)	80	72			79	80			77	82			80	77		
Sust. Fish	Low (%)	10	12	0.5	0.79	11	10	0.3	0.85	12	8	12.3	0.11	8	17	11.5	0.02
	Med (%)	26	22			25	25			28	22			25	27		
	High (%)	64	66			64	65			60	70			68	57		
Food Waste	Low (%)	5	6	4.0	0.13	6	5	0.2	0.90	7	3	11.8	0.03	3	10	13.1	0.01
	Med (%)	21	11			19	20			22	15			19	20		
	High (%)	74	83			75	75			71	81			78	70		
Organic	Low (%)	36	37	0.1	0.95	37	35	4.2	0.12	40	31	11.7	0.15	30	49	16.4	< 0.001
	Med (%)	37	37			34	43			35	39			39	31		
	High (%)	27	26			29	22			25	30			31	20		

Participants over 35 years old generally perceived the recommendations to have a larger benefit than younger participants, although this finding was only significant for ‘reducing food waste’ (*p* = 0.03). The exception was for behaviours relating to meat consumption as younger participants were significantly more likely to perceive the recommendations to ‘prioritise plant-based proteins’ (*p* = 0.04) and ‘limit red and processed meat’ (*p* = 0.01) to have a high environmental benefit. Several gender differences were observed. A significantly higher proportion of women perceived a large environmental benefit with the recommendations to ‘reduce food waste’ (p = 0.01), ‘choose organic produce’ (*p* < 0.001), ‘choose sustainable fish’ (*p* = 0.02) and ‘consume seasonal fruit and vegetables’ (*p* = 0.05). The biggest difference was observed for ‘choose organic produce’, with 50% of males perceiving this behaviour to have a low environmental benefit compared with only 30% of females.

At least three quarters of participants were already in action and maintenance (A/M) stages of change for the sustainable dietary behaviours ‘avoid excess packaging’, ‘limit red and processed meat’, and ‘reduce food waste’ (see Table 4). Around a third of participants were either not interested in, or only thinking about (precontemplation/contemplation stages of change), adopting the recommendations to ‘prioritise plant-based proteins’, ‘reduce consumption of air freighted foods’, and ‘choose organic produce’.

**Table 4:** The number (n) and percent (%) of respondents in precontemplation or contemplation (PC/C), preparation or relapse (P/R), and action or maintenance (A/M) for the sustainable dietary behaviours

	PC/C		P/R		A/M	
	n	%	n	%	n	%
Avoid excess packaging	12	3	95	22	335	76
Buy locally grown produce	44	10	178	40	220	50
Consume seasonal fruits and vegetables	80	18	95	22	267	61
Limit red and processed meat	85	19	24	5	333	75
Prioritise plant proteins	158	36	43	10	241	55
Reduce consumption of air freighted foods	162	37	94	21	186	42
Choose sustainable fish	107	24	54	12	281	64
Reduce food waste	19	4	38	9	385	87
Choose organic produce	142	32	137	31	163	37

Table 5 shows the prediction of stage of change based on demographic characteristics and food choice motives for the sustainable dietary behaviours ‘prioritise plant-based proteins’, ‘choose organic produce’, ‘choose sustainable fish’, and ‘limit red and processed meat consumption’. The food choice motives included in the model were health, cost, environment and taste as these were the most commonly reported factors. The odds ratio (OR) represents likelihood of an individual being in the precontemplation/contemplation (PC/C) or preparation/relapse (P/R) stages of change compared to the reference, A/M stages of change. For three of the four dietary recommendations, none of the factors in our model were significant predictors of a participant being in P/R compared to A/M stage of change. The only exception was for the behaviour ‘choose organic produce’ were individuals over 35 years old (*p* = 0.03) and those who value ‘cost’ as a food-choice motive (*p* < 0.001) are more likely to be in the P/R than A/M stage of change.

**Table 5:** Multinomial regression results for predicting stage of change from socio-demographic factors and food choice motives

	PC/C			P/R			PC/C			P/R		
	β	OR	p	β	OR	p	β	OR	p	β	OR	p
	***Prioritise plant-based proteins***					***Choose organic produce***						
Age < 35 yrs	−0.21	0.81	0.38	−0.09	0.92	0.81	0.71	2.03	0.01	0.58	1.79	0.03
Higher education	−0.33	0.72	0.32	−0.60	0.55	0.18	−0.07	0.94	0.85	−0.33	0.72	0.34
No children	− 0.23	0.79	0.36	−0.50	0.60	0.17	0.09	1.10	0.75	−0.39	0.68	0.16
Female	− 0.62	0.54	0.01	−0.08	0.92	0.83	−0.44	0.64	0.10	−0.13	0.88	0.64
Health	−0.31	0.74	0.29	0.31	1.36	0.51	0.30	1.35	0.36	0.12	1.13	0.70
Environment	−1.19	0.30	< 0.001	−0.46	0.63	0.28	−0.65	0.52	0.04	0.31	1.37	0.32
Taste	0.49	1.63	0.07	0.33	1.39	0.42	0.22	1.25	0.45	0.14	1.15	0.63
Cost	0.53	1.70	0.06	0.42	1.52	0.30	0.92	2.51	< 0.001	1.33	3.77	< 0.001
	***Choose sustainable fish***					***Limit red & processed meat***						
Age < 35 yrs	0.90	2.45	< 0.001	0.36	1.44	0.27	−0.33	0.72	0.27	0.20	1.22	0.68
Higher education	−1.05	0.35	< 0.001	−0.49	0.61	0.26	−0.59	0.55	0.09	−0.38	0.68	0.53
No children	0.44	1.56	0.13	0.24	1.28	0.49	−0.18	0.84	0.55	−0.33	0.72	0.47
Female	−0.01	0.99	0.96	0.01	1.01	0.98	−0.51	0.60	0.07	−0.40	0.67	0.38
Health	−0.16	0.85	0.61	−0.50	0.61	0.18	−0.90	0.41	0.01	0.20	1.22	0.73
Environment	−0.93	0.39	< 0.001	−0.74	0.48	0.06	−1.62	0.20	< 0.001	−0.19	0.83	0.73
Taste	0.12	1.13	0.68	−0.12	0.89	0.74	0.10	1.10	0.76	0.69	2.00	0.19
Cost	−0.04	0.96	0.88	−0.13	0.87	0.72	0.42	1.52	0.23	0.28	1.33	0.59

Reference category was the action/maintenance (a/M) stage of change (plant proteins *n* = 241, PC/C (*n* = 158) P/R (*n* = 43); organic *n* = 163 PC/C (*n* = 142) P/R (*n* = 137); sustainable fish *n* = 281 PC/C (*n* = 107) P/R (*n* = 54); RP meat *n* = 333 PC/C (*n* = 85) P/R (*n* = 24) *PC/C* Precontemplation/contemplation, *P/R* Preparation/relapse

Reporting ‘environment’ as a food choice motive was a significant predictor of a participant being in A/M stage of change compared to PC/C for all the behaviours. Females are more likely to be in A/M than P/C stage of change for ‘prioritise plant-based proteins’ (*p* = 0.01) and participants who stated ‘health’ as a food-choice motive were more likely to be in A/M for ‘limit red and processed meat’ (*p* < 0.001). Conversely, being older than 35 years old was a predictor of being in PC/C stages of change for ‘choose organic produce’ (p = 0.01) and ‘choose sustainable fish’ (p < 0.001). Having a higher education level was a significant predictor of a participant being in A/M for ‘choose sustainable fish’ (p = < 0.001). Having children at home was not a significant predictor of stage of change for any of the behaviours.

## Discussion

### Study sample

Compared to the UK population, the study sample had a higher proportion of females (66% vs. 51%) [55] and those with a higher education level (85% vs. 50%) [56]. As participants were primarily employed adults, there is limited representation from adults ≥65 years old and children ≤18 years old, who in total represent over a third of the UK population [55].

### Perceived importance and readiness to adopt sustainable diet recommendations

For most of the sustainable diet recommendations studied, at least 50% of participants perceived a high environmental benefit and reported being in the action or maintenance stage of change. Furthermore, over half of participants stated environmental sustainability as an influence on their dietary choices. This indicates that overall, the study participants are aware of the relationship between food and the environment and are engaged with sustainable diet recommendations. Additionally, the proportion of participants in the relapse stage of change was low for all recommendations indicating that if consumers can be influenced to adopt sustainable diet recommendations, behaviour change can be maintained long term. However, it is important to note the potential for social-desirability bias in surveys and that participants’ responses may not reflect their actual purchasing behaviour [57].

The behaviours considered to offer the largest environmental benefit were ‘reduce consumption of air-freighted foods’, ‘reduce food waste’, and ‘buy locally grown produce’. ‘Consume seasonal fruits and vegetables’, ‘limit red and processed meat’, ‘avoid excess packaging’ and ‘choose sustainable fish’ were also perceived to have a high environmental benefit by most participants. Conversely, participants deemed ‘prioritise plant-based proteins’ and ‘choose organic produce’ to have a lower environmental benefit. Studies using LCA to determine GHG emissions [9] and overall environmental impact [24] of foods demonstrate that avoiding air-freighted foods, choosing organic produce and consuming a plant-based diet are the dietary behaviours which have the largest environmental benefit. This highlights that consumers are aware that the food system impacts the environment but may not understand the impact of specific dietary behaviours as their perceptions are not in line with actual environmental benefit, according to these studies.

This finding is not surprising as determining overall environmental impact is complex due to its many facets, for example, organic farming uses fewer resources and has lower direct GHG emissions but agricultural yields are lower resulting in increased indirect GHG emissions due to land-use change and the need to import food to meet demand [58]. Johnson et al. describe the complexities in defining a sustainable diet in terms of health, food security and environmental sustainability and highlight the need for developing metrics and measurement mechanisms for sustainable diets [10]. Food labels (e.g. Fair Trade, Rainforest Alliance, Carbon Footprint) are a useful way to communicate information to consumers who are motivated to make ethical purchases [59] but may be confusing for consumers when there are trade-offs between different aspects of a sustainable diet.

That the recommendations to ‘reduce food waste’ and ‘avoid excessive packaging’ were perceived to have a high environmental benefit is not surprising as there have been recent media campaigns promoting these issues [60–63]. Additionally, most participants were in the action and maintenance stages of change for these behaviours. However, in terms of environmental impact, the relationship between these two behaviours is complex and may have competing outcomes. For example, packaging plays a key role in preventing food waste by preserving and protecting food in transport and storage [64] and only contributes 3% to the total food-related GHG emissions [9]. Therefore, by purchasing foods with less packaging consumers may be inadvertently increasing food waste elsewhere in the supply chain and increasing the overall environmental footprint of the food system.

On the other hand, there are instances when packaging design leads to increased food waste in the home by encouraging over-purchasing (e.g. multipacks, special offers), being difficult to empty fully or due to conservative sell-by dates [50]. There are also a wide variety of packaged foods including fresh produce, meat and fish, dairy products as well as processed foods such as cereals and confectionary. The concept of ‘excess packaging’ is ambiguous and could potentially lead to confusion amongst consumers or avoidance of nutritious foods such as fruits and vegetables which are often packaged. For this reason, it may be prudent not to include avoiding foods with excessive packaging amongst sustainable dietary guidelines but instead to focus on behaviours such as avoiding highly processed foods, only buying the quantity of food required, using left-overs and recycling packaging where possible.

A larger proportion of participants reported that they have already started to adopt the recommendations to ‘reduce food waste’ and ‘avoid excess packaging’, compared to ‘prioritise plant-based proteins’ and ‘choose organic produce’. This suggests that behaviours which require no nutrition knowledge or significant dietary changes are more readily adopted by consumers. O’Keefe et al. [43] also observed that behaviours are perceived more positively when they fit within an individual’s existing competencies. Redesign of packaging to reduce food waste or use of bio-degradable materials is another effective way to reduce the environmental impact of packaging which does not require decision-making or action from the individual [65]. Promotional campaigns and interventions could then focus on improving consumers’ nutrition knowledge and skills to enable them to adopt behaviours which have a larger overall environmental benefit, rather than on avoiding packaging waste.

The results of this study indicate a high level of awareness of the environmental impact of red and processed meat consumption and three-quarters of participants reported that they have already started to limit these foods. On the other hand, a quarter of participants reported being in the pre-contemplation stage of change for this behaviour indicating that there are some individuals who are resistant to reducing meat consumption. Barriers to reducing meat consumption include a strong attachment to the taste and familiarity of meat [36, 66] and the belief that personally reducing meat consumption will not make a significant difference on a global scale [42]. Those with a strong attachment to meat consumption may deny its negative impacts to reduce their cognitive dissonance [67]. For consumers resistant to reducing meat consumption it may be better to promote replacing conventional red and processed meat products with lower environmental impact alternatives such as white meat and sustainable fish which have been shown to be more readily accepted than plant-based protein sources [40]. As almost two-thirds of participants reported choosing sustainable fish some or most of the time and most participants reported consuming fish at least once per week, sustainable fish may be a culturally acceptable alternative to meat.

Only a small proportion of participants reported that they wanted to ‘limit red and processed meat consumption’ and ‘prioritise plant-based proteins’ but there were barriers preventing them. This was a surprising result as several practical barriers to reducing meat consumption have been identified including lack of choice when eating out and difficulty preparing vegetarian meals [37, 40, 53]. As there has been an increase in meat-free options in UK restaurants and supermarkets in recent years as well as campaigns promoting how to adopt a meat-free diet [68, 69] these barriers may be becoming less relevant for UK consumers. However, the potential health and environmental benefits of reducing meat consumption depends what replaces meat in the diet [8]. For example, 46% of participants reported consuming processed meat alternatives at least once per week (Fig. 1) which are not nutritionally comparable with meat and may be missing key nutrients such as iron, zinc and vitamin B12 [70].

Additionally, over 85% of participants reported that they consume nuts, seeds, beans and lentils at least once per week (Fig. 1) but modelling studies calculating the potential reductions in GHG emissions from healthy, sustainable diets have typically not focused on replacing meat with other plant-based sources of protein specially but on a variety of plant-based foods, including fruits, vegetables and cereals as well [8, 19]. Therefore it is not clear what the environmental benefit would be if meat were replaced by these plant-based sources of protein. Rosi et al. compared reported dietary data for omnivores, vegetarians and vegans and observed that meat-free diets generally had a lower environmental impact in terms of water use, land use and CO_2_ emissions but that some vegan participants had an extremely dietary environmental footprint due to only consuming fruits [17]. Therefore, it is important for policy and interventions encouraging sustainable diets to promote consumption of healthy foods which also have a low environmental footprint, rather than solely encouraging consumers to eat less meat.

Despite being perceived as important aspects of a sustainable diet, avoiding air-freighted foods and buying local and seasonal produce appear to be difficult behaviours for consumers to adopt with 30–50% in the contemplation and planning stages of change. Lack of availability of local, seasonal produce may be a significant barrier in the UK as over half of food sold is imported [71]. Knowledge of seasonality of foods is low amongst UK consumers which may be due to supermarkets stocking imported produce all year round [43]. Swedish national dietary guidelines promote traditional foods and a higher proportion of the Swiss population report consuming local and seasonal produce [39, 72]. Conversely, UK consumers are encouraged to eat a Mediterranean-style diet to avoid disease despite many of these foods not being native to the country [73]. The results of the current study therefore suggest a need for exploration of policy to enable motivated consumers to adopt these behaviours, for example promoting local and seasonal produce via dietary guidelines and subsidizing local agriculture.

Government policy also has potential to make organic agriculture more economically viable for farmers [74]. Although choosing organic produce has a significant environmental benefit, it was perceived to have the lowest environmental benefit of all of the recommendations and only a third of participants have started to adopt this behaviour. The higher price and lack of availability in supermarkets have previously been identified as barriers to consumers purchasing organic products [75, 76] therefore, reducing the price may increase willingness to adopt this behaviour amongst consumers who perceive this behaviour to be important. However, a third of participants reported being in the pre-contemplation and contemplation stage of change therefore it is clear that strategies to raise awareness of the environmental impact of conventional farming methods and the benefits of organic farming are also needed.

### Factors influencing perceived importance and readiness to adopt sustainable diet recommendations

Younger participants and those with a higher education level perceived the recommendations to ‘limit red and processed meat consumption’ and ‘prioritise plant-based proteins’ to have a higher environmental benefit, compared to older participants (Table 3), although this did not correspond to being more ready to adopt these behaviours (Table 5). The interest in plant-based diets amongst younger consumers may be explained by the emergence of nutrition and dietary trends within urban areas and celebrity endorsement of meat-free diets [77, 78]. Higher meat consumption has been associated with lower education level and social class [79, 80] and it has been observed that younger consumers associate healthy eating with moral worth and social status [81]. It may also be that adopting a plant-based diet is now perceived as morally superior due to the negative impact of meat consumption on individual heath and the environment [77].

Females associated a higher environmental benefit with all sustainable diet recommendations, although this finding was only significant for ‘consume seasonal fruits and vegetables’, ‘reduce food waste’, ‘choose organic produce’ and ‘choose sustainable fish’. Women are typically more involved with food purchasing and preparation which could lead to them being more aware of food-related issues [36, 82]. Ethical consumption habits are generally considered to be more feminine which could lead to males disregarding these behaviours [83]. Furthermore, female were more likely to report adopting behaviours relating to reducing meat consumption which reflects UK National Diet and Nutrition Survey (NDNS) data [84]. This is not surprising due to the connotations of meat and masculinity [85, 86] therefore overcoming these gender stereotypes is necessary to influence male consumers to adopt sustainable diet recommendations.

Older participants were more concerned about the impact of food waste, which has previously been attributed due to generational rather than age-related differences in food-habits and values [38]. Studies of food waste highlight that the main reasons for generating food waste amongst younger consumers are concerns over freshness [87] improper storage and excessive purchasing due to more frequent shopping and retail marketing strategies [88, 89]. Older consumers may also have greater skill and knowledge to plan meals and use leftover food [88, 90]. Therefore, both raising awareness of the impact of food waste amongst younger consumers as well as knowledge and skills for reducing food waste is important. On the other hand, those ≥35 years old were twice as likely to be in the pre-contemplation and contemplation stages of change compared to action and maintenance for ‘choose organic produce’ and ‘choose sustainable fish’, indicating that younger generations are more engaged with these behaviours. Purchasing organic food and sustainable fish have been associated with presentation as a ‘green consumer’ which is more prevalent amongst younger consumers, particularly those with a higher education level which is reflected in our findings [91, 92]. This is an interesting topic which could be explored further as there may the potential to develop ‘green consumer’ role models which could also appeal to older consumers.

### Study limitations

This study employed a self-recruit sampling method which has the limitation that those with an interest in the topic may be more inclined to take part [93]. Additionally, the study recruited participants primarily from the Environment Agency employees who may be more aware of sustainability issues than the general public due to the nature of their employment. Our sample included a higher proportion of females and university educated individuals which is a common limitation of studies in this field. The sample was also concentrated in several urban regions of England, therefore is not representative of consumers living in rural areas or in other parts of the UK.

Additionally, aspects of the study are based on participant’s reported dietary intake, food choice motives and behaviours which may not reflect their actual actions. The methods employed in this study, particularly the stage of change construct, are suitable for meeting the objective of assessing consumers’ readiness to adopt sustainable dietary recommendations but may not represent actual purchasing behaviour. Previous studies have highlighted that self-reported dietary data can be used to inform public guidance and policy [94], however adjustment is required to account for under-reporting [95]. It would therefore be beneficial for future studies to analyse actual consumer food purchases and use alternative methods of measuring dietary intake such as 24-h recall or food diary assessments.

## Conclusions

The conclusions of this study are that participants are aware of the environmental benefit associated with adopting various sustainable dietary recommendations, although they may not fully understand which behaviours offer the largest environmental benefit. The results indicate that dietary guidelines and campaigns promoting sustainable diets should focus on raising awareness of the environmental benefit of prioritising plant-based proteins and choosing organic produce rather than packaging and food waste as consumers are already aware of these issues. However, these findings are limited to the study sample which is not representative of all UK consumers. Further research is therefore recommended to target other population groups such as older consumers, those in rural communities or manual work as well as consumers in other parts of the UK.

A high level of engagement with sustainable diet recommendations was observed overall which indicates that dietary guidelines incorporating sustainability aspects may be well-accepted by consumers within our sample, although further research would be needed to confirm this. Conversely, several behaviours were identified that participants were ready to adopt but were unable to do so, such as buying local and organic produce and reducing consumption of air-freighted foods. This finding suggests a need for further governmental policy and industry action to reduce some of the barriers associated with buying sustainably produced and transported foods. Further research is recommended to explore these barriers and potential solutions.

Differences in perceptions and reported behaviours were observed between gender, age and education groups. This data could be used as a starting point for further research into this topic or to identify potential target groups for future campaigns and interventions addressing environmentally sustainable diets.

## Supplementary Information

**Additional file 1.**

## Data Availability

The datasets used and/or analysed during the current study are available from the corresponding author on reasonable request.

## Abbreviations

A: Action (stage of change)
BDA: British Dietetic Organisation
C: Contemplation (stage of change)
DEFRA: Department for Environment, Food and Rural Affairs
FAO: Food and Agriculture Organisation
GHG: Greenhouse gas
LCA: Life cycle analysis
M: Maintenance (stage of change)
OR: Odds ratio
P: Preparation (stage of change)
PC: Pre-contemplation (stage of change)
PHE: Public Health England
R: Relapse (stage of change)
